# Managing Directors’ Perspectives on Digital Maturity in German Hospitals—A Multi-Point Online-Based Survey Study

**DOI:** 10.3390/ijerph19159709

**Published:** 2022-08-06

**Authors:** Anja Burmann, Burkhard Fischer, Nico Brinkkötter, Sven Meister

**Affiliations:** 1 Chair of Health Informatics, Faculty of Health, School of Medicine, Witten/Herdecke University, 58455 Witten, Germany or; 2 Department Healthcare, Fraunhofer Institute for Software and Systems Engineering, 44227 Dortmund, Germany; 3 Krankenhausgesellschaft Nordrhein-Westfalen e. V, 40237 Düsseldorf, Germany

**Keywords:** digital maturity, digital hospital, managing director, Hospital Future Act

## Abstract

Background: The digitalization and integration of data are increasingly relevant for hospitals. Several methods exist to assess and structurally develop digital maturity. However, it is notable that German hospitals lag behind the European average with respect to digitalization. Objective: We hypothesized that: (a) the perspective of hospital managing directors regarding the state of digitalization in German hospitals plays an important role in the investigation of barriers, and (b) the Hospital Future Act in 2020 may help to surmount those barriers. Methods: Aligned with the Checklist for Reporting Results of Internet E-Surveys (CHERRIES), two online surveys were conducted, one in 2019 and one in 2021. Results: The first study covered 184/344 hospitals and the second, 83/344. The responsibility for deciding on the implementation of digitalization lay with the management (115/184; 62.5%). About 54.9% (101/184) of the managing directors desired digitally supported workflows, together with employees or users. In total, 74.7% (62/83) of hospital managing directors expressed an increase in digitization compared to 2019, with a percentage increase of 25.4% (SD 14.41). In some cases, we analyzed the data using an ANOVA, chi-squared test and Pearson’s correlation, but there was no significant relation identified among the variables. Conclusions: This online-based survey study demonstrated that the development of a digitalization strategy is still strongly tied to or dominated by the attitude of the management. One could assume a lack of acceptance among employees, which should be surveyed in future research. The Hospital Future Act, as well as the COVID-19 pandemic, has positively influenced the digital maturity of hospitals.

## 1. Introduction

In an aging population, the prevalence of multimorbidity increases; therefore, the required variability of treatment pathways rises as well [1]. At the same time, this increases the treatment costs, while the market share of healthy health-insurance contribution payers declines. Growing process complexity and rising costs lead to the necessity of increasing efficiency in healthcare processes. There have been initiatives aiming for centralized decision-making, increased harmonization and the standardization of medical processes, which is a reasonable pathway for ensuring treatment quality and patient safety [2].

### 1.1. Digitalization in German Hospitals

In order to cope with the challenges that the healthcare domain is facing, process efficiency and the utilization of resources is getting more and more important. The timely availability of relevant information at the relevant location to the right people represents the keystone of an efficient hospital [3]. Thus, digitalization and the integration of data seem to be increasingly relevant for independent healthcare-providing organizations, as well as for streamlining intersectoral healthcare provision. However, the adoption of digitalization within healthcare institutions throughout the world is still ongoing [4]. Various assessment tools, called maturity models (MMs), are used to formalize the status of digitalization. They enable the depiction of the current status of development of an organization, a process or a structure with differing focuses [5]. The general concept of modeling maturity stems from the field of software development [6]. This concept has hitherto been transferred into numerous approaches in multiple domains, in order to incorporate specific characteristics and conditions or to address a particular aspect [5,7]. Since healthcare provision relies heavily on process dynamics, interprofessional collaboration and human-centeredness [8], several MMs, particularly in this field, have been suggested as well [9,10,11]. The focus areas vary from the maturation of a picture archiving and communication system [12], via the ability to adopt IT innovations [13] or to manage a personal health record [14], to the usage of data analytics [15]. Several authors, however, reported the lack of practical applicability of these solutions [16,17]. Burmann et al. [18] argue that the structural development of the digitalization of a hospital, using a maturity measurement tool, requires a multi-perspective approach. This implies not only the readiness of technology but also the readiness of processes and the humans involved. At the same time, a digital strategy is required, as well as increasing the digital literacy or digital competency of employees and patients. The approach of Burman et al. integrates several aspects into one assessment.

One commonly adopted method to objectify and benchmark the degree of digitalization of hospitals is the electronic medical record adoption model (EMRAM) provided by the Healthcare Information and Management Systems Society (HIMSS) [19,20,21]. The EMRAM is an eight-stage (0–7) maturity model that measures the use of digital technology to achieve a paperless environment. More than 2500 hospitals in Europe have been assessed using EMRAM. With respect to Germany, Klauber et al. evaluated the EMRAM score of 164 German hospitals, collected between 2014 and 2017, and calculated a mean of 2.3 (EU mean 3.6) [22]. Later, they revised the assessment criteria and created a new database, for which no comprehensive analyses have been published as yet. The regularly published Healthcare IT Report [23,24,25] reflects a similar trend for German hospitals: 59.5% of the respondents stated that data transfer across different departments within the hospital (in this case, from the operating room to the intensive care unit) happens digitally, while 81% of the respondents stated that data transfer to resident doctors is still a paper-based process [25].

### 1.2. Digitalization throughout Europe

An international comparison by the Bertelsmann Stiftung confirmed a heterogeneous degree of digitalization across hospitals in Germany [26]. The aforementioned international analyses also showcased the lead held by other European countries when it comes to digitalizing healthcare [26]. In particular, Scandinavia and the Baltic countries show the impact of digitalization on efficiency and efficacy in the public sector in general [27,28]. Scandinavian countries are structurally and holistically driving the digital transformation of healthcare by following national strategies, which affect not only all aspects of public life but also the healthcare provision sector [29]. Some of the most digitally advanced hospitals can be found in Denmark. The country started to establish so-called “super-hospitals” as part of the Danish digitalization strategy [30,31]. These hospitals offer centralized and highly specialized healthcare services across the country, which leads to a high number of patients at each of these super-hospitals. Technologically, the strategy supports a high volume of patient flow through a hospital via an encompassing and fully digitalized workflow. Therefore, Danish super-hospitals are entirely equipped with digital technology for tracking and tracing, the digital support of diagnostics and treatment and telemedicine, as well as automated documentation, resulting in a paperless organization [32]. The Danish success story in digitalizing their hospitals is certainly favored by the central orchestration and financing of this project in the five Danish regions. However, management-level decision-making has strongly driven the digitalization progress in Denmark, not only from national but also from regional and local instances and actors. This leads to the question of what structural impediments influence the progress of German hospitals with respect to digitalizing their services, and what role hospital managers play in this process.

### 1.3. Hospital Future Act and Digital Radar

Due to the significant digitalization backlog, which, among other things, is caused by a long-lasting investment backlog, a law for the structural and financial support for health provision was enacted in 2020 with the Hospital Future Act. A total of EUR 4.3 billion of public funding is available for increasing the digital maturity of German hospitals. Funding is provided for eleven key areas, known as objects of funding (Fördertatbestand FTB), e.g., decision support (FTB 4) or IT and cybersecurity (FTB 10).

In addition, the so-called DigitalRadar, an EMRAM-based tool for measuring the digital maturity of hospitals, was defined by the authors of [33]. This is a self-assessment tool used by the hospitals, the completion of which is a mandatory part of the application process for funding. Initial results calculated an average value of 33.25 (SD 10.18, max 100) [34].

### 1.4. Objectives

The study is based on the aforementioned status quo of the digital maturity of German hospitals, the lower degree of digitalization in Germany in comparison with other European countries, and the prospects offered by the Hospital Future Act, since:

1. Digitalization comes through digital transformation. Transformational processes in a company are a central management task for which the management is jointly responsible. However, the attitude of hospital management bodies in Germany toward digital transformation is unclear. A look at Denmark showed that the decisions were made at the management level. Therefore, we hypothesized that there has to be a multi-dimensional perspective on the part of managing directors regarding the situation of digitalization, or rather the maturity of German hospitals, in order to obtain a realistic, hospital-oriented viewpoint on the barriers hindering German hospitals in digitalizing their systems. While Scandinavia has advanced further in digitalizing the public sector in general, it is notable that German hospitals lag behind the European average with regard to digitalization. Since the German hospital sector is organized federally and is self-governed, the hospital managers are required to adopt the role of the institutional prime mover, regardless of their own digital affinity.
2. One frequently mentioned reason for the low level of digitalization is the lack of financial resources for investment, which leads to an enormous investment backlog [35,36,37]. With the announced legal amendments to the Hospital Future Act, this factor would become relative. However, there is currently a lack of research describing the change perceived by the managing directors as a result of the Hospital Future Act. This means one has to go beyond the status quo mentioned above, under (1), to survey the changes that are assumed.

In this analysis, the authors are seeking to contribute to the following research objectives:

- Objective O1: The objectification of managing directors’ perception of the digital maturity of German hospitals regarding their technological and organizational status.
- Objective O2: Investigation of the managers’ anticipation of the future role of digitalization in their hospital.
- Objective O3: Identification of barriers from a managerial perspective, which are hindering German hospitals in their progress toward digitalization.
- Objective O4: Survey of digital transformation perceptions from 2019 to 2020, under the influence of the Hospital Future Act.

In order to achieve these objectives, the presented article is organized as follows: Section 2, “Materials and Methods” outlines the study design, respondent recruitment, data handling and data analysis. Section 3, “Results”, presents the survey responses, as well as the relevant analyses between answers. Following that, Section 4, “Discussion”, summarizes the principal results and discusses the study’s limitations, conclusions, and the potential for future research.

## 2. Materials and Methods

The following work was conducted as a multi-point study. The first study was conducted in 2019 and the second study in 2021 after the announcement of legal changes to promote digitalization called Krankenhauszukunftsgesetz (Hospital Future Act).

The target cohort of the studies was the managing directors of German hospitals. According to a national survey done by Alten et al. [38] the characteristics of the population of managing directors can be described as follows: Hospitals have an average of 2.5 managing directors, 87% of whom have a degree in business administration. The average age is 52 years and 81% of the managing directors are male. They tend to change their employer very often,, mostly from internal disputes and economic failure.

Due to operational management obligations and the strongly performance-based remuneration system for the achievement of objectives, the target group’s time availability is severely limited. In addition, the average age range shows that the target group does not belong to twenty-first-century “digital natives”. Thus, gaining access to this group is not trivial, which is why the study’s design, recruitment and completion were carried out in collaboration with the Krankenhausgesellschaft Nordrhein-Westfalen (KGNW). The KGNW is the representative body of 344 hospitals within the region of North Rhine Westphalia and is part of the German Hospital Federation, with 1903 member hospitals. In order to incorporate representatives of the respective group, the authors set up a web-based investigation, following the recommendations for conducting web-based surveys from Dillman et al. and Schleyer and Forrest [39,40]. The development process started with (1) identifying relevant questions through a literature review and cross-validating the survey within an expert panel, (2) carrying out a pre-test and (3) implementing and executing the final online survey.

We implemented the surveys using the LimeSurvey tool, which ensured cross-browser compatibility. LimeSurvey Community Edition is a free and open source on-line statistical survey web app distributed under the GNU General Public License by LimeSurvey GmbH. There were no more than three questions displayed per web page. With respect to the small number of questions, we decided against adaptive questioning. The survey was voluntary and anonymous, and the target population was defined as the group of hospital managing directors from the KGNW. The presented report of the methodology and results also takes into consideration the the Checklist for Reporting Results of Internet E-Surveys (CHERRIES) for reporting e-survey results, as suggested by Eysenbach [41] (see Table A3).

### 2.1. First Study

The study took place in 2019 for a period of 3 months, including pre-testing. The home page of the online survey contained a welcome message, further details on the study and information about anonymous data handling. The questionnaire development phase resulted in a two-tier questionnaire, with part A covering the 13 main items and part B containing four items requesting demographic information about the hospital. Regarding part A, two of the thirteen items were single-choice closed-ended questions (A2, A3), four items were designed as a graphic rating scale, with verbally labeled endpoint categories (A1, A5, A10, A11), and the rest of the items were closed-ended multiple-choice questions (A4, A6, A7, A8, A9, A12, A13). The demographic parameters included hospital size according to the number of beds, the number of in- and out-patients treated per year and the type of sponsorship of the hospital (public, non-profit or private). The language of the questionnaire was German. The presented questions are summarized in Table A1, translated into English.

### 2.2. Second Study

The second study was executed in 2021 for a period of 4 months, including pre-testing. After being introduced with a welcome message, the online survey tool informed the respondent about the privacy policy and required the consent of the participant to proceed. The questionnaire covered seven main items (A to G) and four demographic items (H to K). Most items were single-choice-closed-ended questions. Item F was a closed-ended multiple-choice question.Item B can be described as a rating scale from 0 to 100. The demographic parameters were similar to those from the first study. The language of the questionnaire was German; the presented questions are summarized in Table A2, translated into English.

### 2.3. Recruiting (Both Studies)

To ensure high-quality responses, we decided against snowball sampling or passive recruiting and instead recruited the participants actively in three stages: first, we announced the study one month in advance, by sending a personal letter by mail to all the 344 managing directors of the KGNW member hospitals. Second, we used the same procedure to inform them about the start of the study. Third, we reminded all managing directors by email two weeks before the end of the study. The letter, as well as the email, contained a link to the online survey. To prevent multiple voting, in the second step, we attached a unique one-time token for every participant. Thus, we also prohibited voting from other externals, to impede statistical noise. We assumed that all representatives of the population in their professional roles had access to the Internet. An under-representation of specific groups was not anticipated, while a self-selection bias was discussed [42]. However, this effect is not considered to be particularly high; as described above, management consists of an average of 2.5 people per organization. The authors assumed that due to political developments, interest in the queried topic could be presupposed.

### 2.4. Data Privacy (Both Studies)

The survey system handled the responses anonymously. In order to manage the survey reminder, as well as to prevent multiple participation, we used a personalized token to enable access restriction. The authors created the token list in LimeSurvey. Our research partner, Fraunhofer ISST, operated the survey system on a dedicated server. By adopting the anonymity feature in LimeSurvey, the survey system handled token lists and datasets that were technically separated from each other. In addition, it did not track the IP addresses. The KGNW acted as the trust authority, handling the tokens and invitations sent to the participants during the study. Thus, no researcher was able to map results to a specific hospital. After closing the survey, the KGNW deleted the interconnection of tokens and participants.

### 2.5. Data Analyses (Both Studies)

All collected data were transferred into R (Version 4.2.X) and were analyzed using descriptive statistics [43]. The authors investigated the ratio scales for correlation, using Pearson’s correlation coefficient, the chi-squared test and Fisher’s exact test, as well as an analysis of variance (ANOVA). The results of the single-choice questions were coded and single factor variance analyses were carried out, looking for differences between the groups regarding the self-assessed degree of digitalization. The results for the multiple-choice questions were coded as well [44].

## 3. Results

In the following, we will provide the results of the first study, conducted in 2019 and, below, those of the second study, executed in 2021.

### 3.1. Study 1

In total, 231 of the requested actors from 344 hospitals started the survey by using their personalized token. A dropout rate of 20.3% (47 incomplete participations) led to 184 incorporable data sets. Thus, we achieved a response rate of 53.5% from the group of managing directors who were associated with the KGNW (184/344). The basic details regarding the sample are shown in Table 1. It roughly represented the shares of hospital sizes according to the number of beds across the nation, apart from the smallest category, which was underrepresented in this study. The number of inpatient cases was more or less equally distributed, at between 5000 and 30,000 cases. In contrast, the number of outpatient treatments was predominated by hospitals with more than 30,000 cases (63/184, 34.3%).

In the first question (A1), we examined the estimated degree of digitalization of the hospitals, on a scale from 0% to 100%, whereby 100% meant that paper-based processes had been completely replaced by digital processes. The mean was 47.1% (SD 18.68), with a maximum of 93% (2/184) and a minimum of 10% (3/184). The wide range of 83% is an expression of the large differences in the implementation of digitalization between hospitals. The estimated degree of implementation of an electronic patient record (A11) (mean 50.8%, SD 27.04), with a minimum of 0% and a maximum of 100%, positively correlates with the general estimation of the digitalization degree (Pearson’s *r* = 0.65, *p* < 0.001).

An ANOVA indicated that there were no significant differences between the sizes of hospitals (B1), established by the number of beds, regarding the general degree of digitalization estimated in A1 (*F*_4,160_ = 2.06, *p* = 0.089). However, the sponsorship of hospitals (B4) showed differences with regard to A1. A classification following the work of Cohens [41] showed a small effect size of η^2^ = 0.04. The direct comparison of pairs via the Scheffe test [42] revealed a significant difference only between the groups “public” (mean 52.9% in A1) and “non-profit” (mean 44.6% in A1; *F*_2,162_ = 6.16, *p* = 0.048). The classification according to the number of cases (both in- and out-patients) showed no differences in their size groups with regard to A1.

A closer look at the different areas of a hospital (A10) showed that work in the wards is still the least digitally supported among the categories queried, with a mean of 47% (SD 21.39; Pearson’s *r* = 0.68, *p* < 0.001), followed by work in the functional areas (mean 56.1%, SD 20.26; Pearson’s *r* = 0.60, *p* < 0.001), purchasing and materials administration (mean 65.1%, SD 21.02; Pearson’s *r* = 0.32, *p* < 0.001) and patient accounting/business management (mean 74.0%, SD 18.04; Pearson’s *r* = 0.30, *p* < 0.001). With respect to the Pearson correlation coefficient, these four areas all positively correlate with the general estimation of the degree of digitalization as presumed.

When asked about the expected added value (A8) gained by digitalizing the hospital, 75.5% (139/184) of the respondents mentioned an improvement in the continuity of care across hospital boundaries and 75% (138/184) mentioned the relief it would offer to employees burdened with repetitive administrative tasks. Only 28.8% (53/184) associated digitalization with cost-saving potential, but 42.9% presumed it could withhold the opportunity to increase the hospital’s attractiveness as an employer (79/184) or its attractiveness for patients (89/184, 48.3%).

In the next question, we asked the managing directors about the main drivers of digitalization initiatives in their institutions (A2). Of the managing directors, 40.2% (74/184) of the respondents stated that the main initiator for digitalization initiatives is the department for corporate development or the executive management board. The second most popular response was that of IT management being the main drivers of change (46/184, 25%). Of the respondents, 22.2% (41/184) named a steering committee consisting of the managing director(s), corporate development, employees and users. These answer groups showed no differences with regard to the general degree of digitalization estimated in A1.

Following this question, the responsibility for deciding on the implementation of digitalization initiatives was queried: in most hospitals (115/184, 62.5%), the responsibility lay with the management. Only a few hospitals were incorporating staff with a dedicated position regarding digitalization in decision-making (24/184, 13%) or a general steering committee (29/184, 15.7%). Interestingly, these answer-groups did not show any differences in the ANOVA with regard to A1, the general degree of digitalization (*F*_5,159_ = 0.41, *p* = 0.841), but also not with regard to A5, the general acceptance of digitalization among employees in the past (*F*_5,177_ = 0.81, *p* = 0.546).

Question A7 queried which areas in the respondent’s hospital already offered digital services to their patients. Responses to this question highlighted that the largest proportion of hospitals offered no digital services to patients at all (104/184, 56.5%). The most widespread digital services available were stated as being in the appointments and patient entertainment areas (52/184, 28.2%), followed by treatment-specific offers of information (35/184, 19%). Only 1.6% (3/184) of the hospitals provided patients with direct access to personal data. As we also gave the respondents the opportunity to enter text in a free-text field, the three most common entries were (1) patient WiFi, (2) a patient-centered website, and (3) patient surveys and complaints management. These results were remarkable since a large number (123/184, 66.9%) of the managing directors perceived the patient as an actor who increasingly demands transparency throughout the treatment process. With respect to information about therapy and diagnostics, the patient is viewed as a receiver of medical information in digital hospitals (127/184, 69%). With the rising availability of smart devices such as fitness trackers, more than one-third of the participants expected the patient to be increasingly a provider of health-related data (71/184, 38.6%).

While patient orientation in terms of specific digitization still differs from the expectations of the future role of the patient in a digital hospital, an increased involvement of employees in digitization initiatives can be seen (A4). About 54.9% (101/184) of the surveyed managing directors designed digitally supported workflows, together with employees and users. Still, 46.7% (86/184) of the surveyed managing directors involved users in the decision-making process, while 34.8% (64/184) incorporated the employees only passively by sharing information about and throughout the whole process. The most common way to involve employees in the process of digital transformation was through the implementation of training programs (111/184, 60.3%).

Based on these answers, the respondents were asked, on a scale from 0% to 100%, to assess how open-minded their own employees had been in the past, in terms of redesigning their work environments and workflows through digitalization projects. Surprisingly, more than half of the managing directors stated that employees were open to digitalization (mean 59.55%, SD 18.29), with a maximum of 96% and a minimum of 10%. Naturally, this correlates with the general degree of digitalization (Pearson’s *r* = 0.35, *p* < 0.001). However, no significant correlation was found by calculating the η-coefficient [43] for each option, related to the general degree of digitalization (A1).

A central question (A13) of the survey was related to the managers’ assessments of the greatest obstacles that they saw in digitalizing their hospitals. A majority of 76% (140/184) of the managing directors saw the lack of financial resources and inadequate standardized infrastructures/interfaces (70/184, 38%) as the greatest obstacles to the implementation of digitalization. The previously mentioned skills shortage was perceived as a large problem in the German health system (67/184, 36.4%), as well as over-regulation by the legislator (60/184, 32.6%).

Eventually, the survey inquired (A12) whether the respondents considered themselves ready to face the challenges of digitalization. Of the respondents, 56.5% (104/184) of the managers were cautiously confident (“It is challenging, but solvable with effort”), while 54.3% (100/184) of the respondents were skeptical about the challenges, due to the necessity of making significant investments to counteract the already existing technological deficit.

### 3.2. Study 2

For the second study, the hospitals were approached in the same way as for the first study. A total of 83 data sets were included in the analysis. This corresponds to a response rate of 24.13% (83/344). The lower response rate, compared to the first study, can be explained by the high burden placed by the COVID-19 pandemic on hospital management. Basic details about the sample (the number of beds, ownership and inpatient/outpatient treatment) are shown in Table 2.

Table 3 shows the frequency and the relative percentages of items A, C, D, E and G. It can be seen that 74.7% (62/83) of hospital CEOs expressed an increase in digitalization since 2019 (Item A), with the basic level of understanding of digitalization (Item D) having changed for 50.6% (42/83) of hospitals. Similarly, 51.81% (43/83) of respondents said that they have changed their structures and approaches to digitalization (Item E). As shown in Table 4 for item B, the mean value of the percentage increase was 25.4% (SD 14.41). The COVID-19 pandemic (Item C) was mostly evaluated (59/83, 71.08%) as an accelerating event for digitalization. Due to the innovation backlog of hospitals in Germany, a problem that has existed for years, the question as to which goals can be achieved with the resources of the Hospital Future Act was posed (Item G). Only 9.64% (8/83) of respondents stated that the funding was sufficient.

When asked about specific change measures, the CEOs expressed their views as shown in Table 5. The majority of hospitals (53/83, 63.86%) first developed a strategy. It is also evident that measures with a high relation to people, such as ensuring the availability of resources (46/83, 55.42%) and promoting interdisciplinarity (67/83, 44.58%), were considered highly relevant.

We assumed that there might be a correlation between some of the items, e.g., an increase in digitalization should affect the number of approaches or structures used, as well as the understanding of digitalization. Therefore, we applied the chi-squared test and Fisher’s exact test; furthermore, we calculated the contingency matrix. The latter showed several entries that were lower than 5 for the given combinations. Thus, the results have to be interpreted with caution, as we will discuss later in this paper. For items A (Change in the hospital’s level of digitalization since 2019) and D (Change of understanding of digitalization since 2019), where *χ*^2^ (4, N = 83) = 17.07, *p* = 0.00187 could not be verified with Fisher’s exact test (*p* = 0.0992). The same issue happened with A and E (New established structures or approaches since 2019), where *χ*^2^ (6, N = 83) = 18.393, *p* = 0.00532, but *p* = 0.1243 for Fisher’s exact test. Furthermore, we asked about a possible correlation between the size of the hospital (item H) and perceptions with respect to item A, but no significance could be calculated with the chi-squared test (*χ*^2^ (6, N = 83) = 8.9724, *p* = 0.1751).

## 4. Discussion

With respect to the research objectives formulated within the objectives section of this paper, the following findings are of relevance:

1. Managing directors perceive pressure with respect to digitalization. Moving away from paper-based working will be key to fostering the efficiency and efficacy of processes. However, especially in the case of those areas “close to the patient”, such as work on the wards, are only weakly digitalized.
2. Within the hospital of the future, digitalization will help to improve the continuity of care and will help to make the process more transparent to the patient. However, most hospitals do not offer any digital service to the patient at present.
3. The managers mentioned a lack of financial resources as a key barrier to successful digitalization.
4. The COVID-19 pandemic, as well as the established structures through the Hospital Future Act, had implications for the level of digitalization.

In the following section, we will discuss the four key results; the authors believe that, otherwise, the discussion would need a more differentiated format. The online survey underlined the finding that the respondents associated digitalization, on the one hand, with the potential to relieve the workload of employees confronted with an increasing demand for repetitive and automatable tasks. One must treat the terms “efficiency” and “efficacy” with caution when it comes to digitalization. Often, there is no objective data available to prove those statements, as has been discussed by Beaulieu and Bentahar [45] regarding supply chains in healthcare. The efficiency of hospitals was also researched by Kohl et al. [46], who used data envelopment analysis (DEA) but offered the criticism that the validity of quality might be an issue.

An increase in continuity of care for the benefit of the patient could be anticipated. However, a considerable number of the managing directors apprehended obstacles, such as the lack of investment budgets and the lack of infrastructure for continuous information processing, as restraining factors. Sætra and Fosch-Villaronga [47] critically discussed the changing nature of digitalization in healthcare. They introduced a three-level framework in which it is stated that skills and jobs are transforming, but the quality and quantity of care are on different levels. When it comes to health care leadership, its success is all about the 3 Ps: people, processes, and (computer) programs [48].

It is debatable whether the predominance of the lack of financial resources does not overly conceal further challenges. It is clear that at the time of the first study, underfunding and an enormous investment backlog was a major issue [35,36,37]. Assuming that the money would be available, the question arises regarding the areas into which a hospital would invest the money. In addition to strategy orientation, knowledge and skilled personnel are required. Only a few managers mentioned those competencies as a problem. Referring to the work of Kraus et al. [49], they deduced five clusters when it comes to digital transformation in healthcare: operational efficiency by healthcare providers, patient-centered approaches, organizational factors and managerial implications. These five topics were also represented by the items of the survey, but the authors suspect a certain bias here since the provision of financial resources is a necessary precondition; therefore, other answers were deliberately given a lower priority.

We evaluated the involvement of the employees critically. The management mostly controlled digitalization initiatives. Decision-making processes were also highly centralized. Hospitals only realize the broad involvement of employees through training programs. Training seems to be an important point, especially as a way to foster digital competencies; they are missing but are required to create a positive atmosphere [50]. Within our first study, employees were not co-designing partners in digitalization, which could lead to the hypothesis that acceptance of the use of digital solutions could be impaired. Factors affecting this acceptance are job relevance, output quality, result demonstrability, and the perceived ease of use of health information technology [51]. Learning-oriented leadership and adaptive management should be focused upon to emphasize workplace learning, as management strategies are decisive when transforming an organization from health to eHealth [52]. As discussed by Kokshagina [53], the micro-levels (individual levels) and macro-levels (hospital levels) must be aligned through a constructive dialog at the meso-level (department and interdepartmental levels). The alignment of the mindset of relevant stakeholders, behavioral rules and suitable technology are key elements to driving digital transformation [54].

The authors assumed that there is a perception bias, which requires a broader range of questioning for acceptance, taking into account other stakeholders, such as physicians or caregivers. With respect to other work, one could point out that the human factor is a key success factor for the introduction of technology in healthcare [55]. For example, physicians struggling with a given situation will not have the feeling that digitalization increases the efficiency and efficacy of healthcare [56].

Interestingly, we found no significant differences in the estimation of the general degree of digitalization (A1) between hospital categories with respect to size according to the number of beds. Regarding the sponsorship groups, a difference was found between “public” (mean 52.9% in A1) and “non-profit” (mean 44.6% in A1), but not between these two and the group “private”, as one might assume. The “non-profit” group, however, was the group with the lowest mean value in A1. In addition, the different kinds of involvement of employees in the decision-making and implementation processes did not show any significant differences in effect on the self-estimated degree of digitalization, at least not from the managers’ point of view. This is in contrast to the work of Kokshagina [53], as mentioned above. The successful implementation of health information technology would require supporters, change managers, advocates, project managers, decision-makers, facilitators and champions [57].

With regard to the second study, the basic conditions were different. The aforementioned financial restrictions were now countered by the Hospital Future Act, which offers prospective investments of EUR 4.3 billion. Thus, it is to be expected that the perception of the managing directors would turn toward an increase in digitalization. Nevertheless, it must be critically discussed that this could also be influenced by the COVID-19 pandemic. As shown in the second study (item C), 71.08% of the respondents considered that COVID-19 had a positive effect on the degree of digitalization. This effect cannot be sufficiently differentiated on the basis of the data collected, but external studies clearly support this finding [58].

At this point, the authors would like to refer to the correlation analyses of the second study. No significance could be identified since Fisher’s test spoke against the chi-squared test. Nevertheless, it would have been to be expected that, for example, item A and item C should be correlated. A manual examination of the contingency matrix showed that the expected tendency (item A: increased item C: accelerating effect) is recognizable.

### Limitations

First, the authors only considered the perspectives of the managing directors. As described in the presentation of the principal results, there is the presumption of a bias related to particular questions and answers. These limitations would require an extension of the survey context, e.g., the target group. The survey covered only hospitals in the federal state of North Rhine-Westphalia. This plays a role in terms of limitations, at least with regard to the question of the greatest obstacles. The reason for this is the dual financing of hospitals in Germany. The federal states will cover particular investment costs. It is possible that the financial aspect is of less importance in federal states with a higher investment ratio.

Furthermore, we would like to discuss the dropout rate, as well as the response rate. Response rate values ranging from 25–30% are more or less acceptable and one can improve them to as much as 60–70% [59], whereas average rates of around 44.1% are common [60]. Therefore, the response rate of the first study is strong enough to support the external validity of the results. On the other hand, the dropout rate is higher than the usual 10% [61]. As we pre-tested the difficulty of the items, there may have been a conflict with the duration of the survey and time availability. In the second study, the situation is exactly the opposite. We had no dropouts from the survey but only had a response rate of 24.13%, which could be explained by the high burden laid by the COVID-19 pandemic on hospital management from 2020 to 2021.

## 5. Conclusions

Digitalization is an important tool by which hospitals can achieve greater process efficiency. Information continuity through the avoidance of unnecessary paperwork in documentation is a primary goal, but only half of the hospitals included in the study have been able to address this issue (study 1, A1). To what extent the provision of financial resources alone leads to an improvement remains to be critically questioned, as does the lack of early involvement of the employees. However, it is clear that a process of change has begun with regard to digitalization, which may have been forced by COVID-19, as well as by the availability of funding due to the Hospital Future Act. Furthermore, the meso-level between management and employees is still unclear. A large number of the approaches required to initiate change at this level can be assigned to changes in management. Digitalization requires new competencies related to changed or even new processes. Aspects such as the size of the hospital or the sponsorship did not correlate with the perceived status of digitalization. Thus, the authors presume that other variables, such as the presence or absence of a strategy for digital transformation, should be investigated. Furthermore, the willingness of employees to collaborate with digitally supported processes needs to be assessed.

## Figures and Tables

**Table 1:** Basic data describing the sample. B1: number of beds; B4: type of hospital; B2: number of inpatient treatments; B3: number of outpatient treatments.

Item	Response Category	n	%	Item	Response Category	n	%
B1: Number of beds	101–250	49	28.21	B4: Type of hospital	Non-profit	53	63.86
	251–500	66	34.62		Public	24	28.92
	501–1000	46	25.64		Private	6	7.23
	>1000	10	11.54				
B2: Number of inpatient treatments	up to 5000	29	15.76	B3: Number of outpatient treatments	up to 5000	18	9.78
	5001–10,000	34	18.48		5001–10,000	19	10.33
	10,001–15,000	29	15.76		10,001–15,000	21	11.41
	15,001–20,000	33	17.93		15,001–20,000	19	10.33
	20,001–25,000	22	11.96		20,001–25,000	20	10.87
	25,001–30,000	11	5.98		25,001–30,000	24	13.04
	>30,000	26	14.13		>30,000	63	34.24

**Table 2:** Basic data describing the sample.

Item	Response Category	n	%	Item	Response Category	n	%
H: Number of beds	101–250	49	28.21	K: Type of hospital	Non-profit	53	63.86
	251–500	66	34.62		Public	24	28.92
	501–1000	46	25.64		Private	6	7.23
	>1000	10	11.54				
I: Number of inpatient treatments	up to 5000	10	12.05	J: Number of outpatient treatment	up to 5000	9	10.84
	5001–10,000	16	19.28		5,001–10,000	7	8.43
	10,001–15,000	9	10.84		10,001–15,000	11	13.25
	15,001–20,000	14	16.87		15,001–20,000	8	9.64
	20,001–25,000	13	15.66		20,001–25,000	9	10.84
	25,001–30,000	3	3.61		25,001–30,000	9	10.84
	>30,000	18	21.69		>30,000	30	36.14

**Table 3:** Frequencies and relative percentages of items A, C, D, E and G.

Item	Response Category	n	%
A: Changes in the hospital’s level of digitalization since 2019	Increased	62	74.7
	Stagnated	20	24.1
	Decreased	0	0
	Cannot estimate	1	1.2
	No Answer	0	0
C: Impact of COVID-19 on digitalization	None	12	14.46
	Accelerating effect	59	71.08
	Inhibiting effect	6	7.23
	Cannot estimate	6	7.23
	No answer	0	0
D: Changes in understanding of digitalization since 2019	Yes	42	50.6
	No	36	43.37
	No Answer	5	6.02
E: New established structures or approaches since 2019	Yes	43	51.81
	No	8	9.64
	Already established infrastructures	27	32.53
	N/A	5	6.02
G: Sufficient funding through the Hospital Future Act	The level of digitalization in our company is already at a high level, so the focus is on increasing IT security	1	1.2
	The subsidies merely close the investment gap without enabling us to achieve the targeted level of digitalization	72	86.75
	The funding is sufficient to raise the level of digitalization in a meaningful way	8	9.64
	No Answer	2	2.41

**Table 4:** Rating scale item B, expressed as an increase only, with respect to item A.

Item	Response Category	n	Mean	SD	Min	Max
B: Estimated increase/decrease in digitalization	Amount of increase/decrease	62	25.4	14.41	7	65

**Table 5:** Multiple-choice item F, asking for structures and measures to drive digitalization forward.

Item	Category	Yes	No	N/A	Yes %	Full N
F: Structures and measures to drive digitalization forward	Appointment of digitalization officers	16	54	13	19.28	83
	Formation of an interdisciplinary steering committee	37	33	13	44.58	83
	Development of a digitalization strategy/roadmap	53	17	13	63.86	83
	Adaptation of a focus areas of the Hospital Future Act funding items	46	24	13	55.42	83
	Establishment of project portfolio boards	22	48	13	26.51	83
	A contact person for digitization in the departments	18	53	13	21.69	83
	Provision of time and personnel resources	46	24	13	55.42	83

## Data Availability

According to the guidelines for good scientific practice and research data management of Witten/Herdecke University, data can be requested by contacting the corresponding author. Each request is checked by our cooperation partner KGNW and must be approved by them.

## Appendix A

**Table A1:** Translated questionnaire of the first study.

Nr.	Question	Answer Type	Scale Type
A1	How high do you estimate the degree of digitalization of your hospital to be?	rating scale	ratio
A2	Who is the main initiator of digitalization initiatives in your hospital?	single choice	nominal
A3	Who evaluates and decides about the introduction of digital processes and offers for patients and employees?	single choice	nominal
A4	How do you involve employees in the digitalization of their workflows?	multiple choice	nominal
A5	How have your employees accepted the transformation of their work environments/processes through digitalization in past projects?	rating scale	interval
A6	How do you see the role of the patient in the digital hospital?	multiple choice	ordinal
A7	In which areas do you offer digital services to your patients?	multiple choice	nominal
A8	Where do you see added value through digital solutions in hospitals?	multiple choice	nominal
A9	Are processes within your hospital lived as they are defined?	single choice	ordinal
A10	How high do you estimate the digital penetration rate in the individual areas? Wards, functional areas, business administration, materials management?	rating scale	ratio
A11	How far have you come with the introduction of an electronic patient file in your hospital?	rating scale	ratio
A12	In light of current and future developments, how serious do you estimate the danger to be that your hospital should fall behind in digitalization?	multiple choice	nominal
A13	What do you see as the biggest obstacles between you and the self-determined advancement of digitization of your hospital?	multiple choice	nominal
B1	Please rank the size of your hospital according to the number of beds:	single choice	interval
B2	Please indicate the approximate number of cases treated in your hospital per year:	single choice	interval
B3	Please indicate the approximate number of outpatient cases treated in your hospital per year:	single choice	interval
B4	To which sponsorship group does your hospital belong?	single choice	nominal

**Table A2:** Translated questionnaire of the second study.

Nr.	Question	Answer Type	Scale Type
A	How would you rate the change in your hospital’s level of digitalization since the first study in 2019?	single choice	nominal
B	How do you estimate the increase or decrease in digitalization of your hospital?	rating scale	ratio
C	What impact has the Corona pandemic had on digitalization at your hospital?	single choice	nominal
D	Has your understanding of digitalization in hospitals changed since the first study was launched?	single choice	nominal
E	Since 2019, have you established structures or approaches to driving digitalization?	single choice	nominal
F	What structures and measures have you established to drive digitalization forward?	multiple choice	nominal
G	What is your opinion regarding the funding that is now available?	single choice	nominal
H	Please rank the size of your hospital by the number of beds.	single choice	nominal
I	Please indicate the approximate number of inpatient cases treated at your hospital per year.	single choice	nominal
J	Please indicate the approximate number of outpatient cases treated at your hospital per year.	single choice	nominal
K	What is the ownership of your hospital?	single choice	nominal

## Appendix B

**Table A3:** CHERRIES for the first and the second study.

Item Category	Checklist Item	Explanation
Design	Describe survey design	The managing directors of the KGNW formed the target population. At the time of the survey, the KGNW had 344 member hospitals. All of them were located within the federal state of North Rhine Westphalia. The sample is convincing and representative with respect to different demographic parameters (size of hospitals, location urban/rural, patient structure, and so on).
IRB approval and informedconsent process	IRB approval	Ethical review and approval were waived for this study due to the fact that no risks or harm to the participants are to be expected as well as no violation of basic ethical principles, as a result of an internal ELSA evaluation. Furthermore, the authors asked the external advisory board of the KGNW, staffed by hospital managing directors, for approval. The studies were not related to patient treatment or treatment-related processes.
	Informed consent	Participants were informed on the welcome page that it would take approximately 15 min (first study) and 10 min (second study) to complete, that all responses were confidential and anonymous and that reporting would be on an aggregate level only. Consent was indicated when respondents clicked the ‘Go to Survey’ button on this page.
	Data protection	The survey was hosted and all data were stored on its own secure server. No personal information was linked to survey results in any way. The fully de-identified dataset was kept on password-protected computers.
Development and pre-testing	Development and testing	The survey instrument was designed by identifying relevant questions through literature review and cross-validating within an expert panel (five researchers with a focus on digital maturity). The pre-tested questionnaire was conducted with ten managing directors from member hospitals of KGNW. The expert panel approved the final survey.
Recruitment process	Open survey versus closed survey	The survey was closed to the 344 member hospitals of the KGNW.
	Contact mode	All 344 managing directors of the member hospitals were contacted by letter, including a unique code for participation.
	Advertising the survey	The survey was only advertised through invitation, as described above.
Survey administration	Web/E-mail	The survey was hosted on its own web server by the Fraunhofer Institute for Software and Systems Engineering in Germany, using the software LimeSurvey.
	Context	The landing page of the survey was open accessible from an internet page, but, for participation, a token-code was required. Thus, we ensured that only the members of KGNW were able to participate.
	Mandatory/voluntary	The survey was completely voluntary. Users could access the landing page without completing the survey.
	Incentives	No direct incentive was given to the participants.
	Time/Date	Study 1: 2019, 12 weeks Study 2: 2021, 16 weeks
	Randomization of items or questionnaires	Survey items were not randomized.
	Adaptive questioning	There was no need to reduce the amount due to the small number of questions and the approximated period.
	Number of Items	Study 1: 13 main items and four additional items requesting demographic data about the hospital. Study 2: 7 main items and four additional items requesting demographic data about the hospital.
	Number of screens (pages)	One welcome page, two pages with survey items (one page per question group)
	Completeness check	All survey items were deemed to be mandatory, and respondents were prompted to complete outstanding items before leaving the survey page.
	Review step	A “back” button was provided if participants wished to edit previous answers.
Response rates	Unique site visitor	Not relevant, since a closed group was explicitly invited to participate in the survey via a unique token-code. Additionally, no cookies or IP checks were used.
	View rate	Not relevant, since a closed group was explicitly invited to participate in the survey via a unique token-code. Additionally, no cookies or IP checks were used.
	(Ratio unique site visitors/unique survey visitors)	Not relevant, since a closed group was explicitly invited to participate in the survey via a unique token-code. Additionally, no cookies or IP checks were used.
	Participation rate	Not relevant, since no unique site visitors were recorded.
	Completion rate	Study 1: 184/231 = 79.6% Study 2: 84/103 = 81.5%
Preventing multiple entries from the same individual	Cookies used	No, not necessary. A unique token-code was provided.
	IP check	No, not necessary. A unique token-code was provided.
	Log fileanalysis	Not used.
Analysis	Handling of incomplete questionnaires	Only completed questionnaires were included in the final dataset.
	Questionnaires submitted with an atypical timestamp	No “straight-liners” were identified in post hoc tests. All completed datasets were performed within the maximum time minus 6 min.
	Statisticalcorrection	No statistical correction procedures or weightings were used in the analysis.

## References

[1] Ng A., Fong B., Yuen P. (2018). Sustainable Health and Long-Term Care Solutions for an Aging Population.

[2] Friesdorf W., Buss B., Marsolek I. (2007). Patient safety by treatment standardization and process navigation–a systems ergonomics management concept. Theor. Issues Ergon. Sci..

[3] Bardach N.S., Huang J., Brand R., Hsu J. (2009). Evolving health information technology and the timely availability of visit diagnoses from ambulatory visits: A natural experiment in an integrated delivery system. BMC Med. Inform. Decis. Mak..

[4] World Health Organization Global Strategy on Digital Health 2020–2025. https://www.who.int/health-topics/digital-health#tab=tab_1.

[5] Wendler R. (2012). The maturity of maturity model research: A systematic mapping study. Inf. Softw. Technol..

[6] Humphrey W.S. (1988). Characterizing the software process: A maturity framework. IEEE Softw..

[7] Pöppelbuß J., Niehaves B., Simons A., Becker J. (2011). Maturity Models in Information Systems Research: Literature Search and Analysis. CAIS.

[8] Pomare C., Long J.C., Churruca K., Ellis L.A., Braithwaite J. (2020). Interprofessional collaboration in hospitals: A critical, broad-based review of the literature. J. Interprof. Care.

[9] Carvalho J.V., Rocha Á., Abreu A. (2016). Maturity Models of Healthcare Information Systems and Technologies: A Literature Review. J. Med. Syst..

[10] Gomes J., Romão M. (2018). Information System Maturity Models in Healthcare. J. Med. Syst..

[11] Kolukısa Tarhan A., Garousi V., Turetken O., Söylemez M., Garossi S. (2020). Maturity assessment and maturity models in health care: A multivocal literature review. Digit. Health.

[12] Van de Wetering R., Batenburg R. (2009). A PACS maturity model: A systematic meta-analytic review on maturation and evolvability of PACS in the hospital enterprise. Int. J. Med. Inform..

[13] Esdar M., Liebe J.-D., Weiß J.-P., Hübner U. (2017). Exploring Innovation Capabilities of Hospital CIOs: An Empirical Assessment. Stud. Health Technol. Inform..

[14] Pak J., Song Y. (2016). Health Capability Maturity Model: Person-centered approach in Personal Health Record System. Surfing the IT Innovation Wave, Proceedings of the 22nd Americas Conference on Information Systems (AMCIS 2016), San Diego, CA, USA, 11–14 August 2016.

[15] Sanders D., Burton D., Protti D. (2013). Healthcare Analytics Adoption Model: A Framework and Roadmap. https://www.healthcatalyst.com/white-paper/healthcare-analytics-adoption-model/.

[16] Blondiau A., Mettler T., Winter R. (2016). Designing and implementing maturity models in hospitals: An experience report from 5 years of research. Health Inform. J..

[17] Burmann A., Meister S. Application of Maturity Models in Healthcare: Findings from Multiple Digitalization Case Studies. Proceedings of the 14th International Joint Conference on Biomedical Engineering Systems and Technologies.

[18] Burmann A., Deiters W., Meister S., Pfannstiel M.A., Da-Cruz P., Mehlich H. (2019). Digital Health Maturity Index: Analyse des Digitalisierungsgrades im Krankenhaus. Digitale Transformation von Dienstleistungen im Gesundheitswesen VI.

[19] Junger D., Kloos U. (2017). Analyse von Reifegradmodellen zur Unterstützung der Digitalisierung von Krankenhäusern. Informatics Inside: Digital Future: Informatik-Konferenz an der Hochschule Reutlingen, 10. Mai 2017.-(Informatics inside; 17).

[20] Van Poelgeest R., van Groningen J.T., Daniels J.H., Roes K.C., Wiggers T., Wouters M.W., Schrijvers G. (2017). Level of Digitization in Dutch Hospitals and the Lengths of Stay of Patients with Colorectal Cancer. J. Med. Syst..

[21] Ayat M., Sharifi M. (2016). Maturity Assessment of Hospital Information Systems Based on Electronic Medical Record Adoption Model (EMRAM)—Private Hospital Cases in Iran. IJCNS.

[22] Klauber J., Geraedts M., Friedrich J., Wasem J. (2019). Krankenhaus-Report 2019.

[23] Hübner U., Liebe J.-D., Hüsers J., Thye J., Egbert N., Hackl W., Ammenwerth E. (2015). IT-Report Gesundheitswesen: Pflege im Informationszeitalter.

[24] Hübner U., Esdar M., Hüsers J., Liebe J.-D., Rauch J., Thye J., Weiß J.-P. (2018). IT-Report Gesundheitswesen: Wie Reif Ist Die IT in Deutschen Krankenhäusern?.

[25] Hübner U., Esdar M., Hüsers J., Liebe J.-D., Naumann L., Thye J., Weiß J.-P. (2020). IT-Report Gesundheitswesen: Wie Reif Ist Die Gesundheits-IT Aus Anwenderperspektive?.

[26] Thiel R., Deimel L., Schmidtmann D., Piesche K., Hüsing T., Rennoch J., Stroetmann V., Stroetmann K. (2018). Smart Health Systems: International Comparison of Digital Strategies.

[27] Stavytskyy A., Kharlamova G., Stoica E.A. (2019). The Analysis of the Digital Economy and Society Index in the EU. Balt. J. Eur. Stud..

[28] Möller T., Grinblat Etterer D., Plugmann P. (2022). Durchbruch KHZG?–Das Krankenhauszukunftsgesetz vor dem Hintergrund strategischer Ansätze zur Digitalisierung der (klinischen) Versorgung in Schweden und Dänemark. Innovationen Im Gesundheitswesen: Rechtliche und Ökonomische Rahmenbedingungen und Potentiale.

[29] Randall L., Berlina A. (2019). Governing the Digital Transition in Nordic Regions.

[30] Healthcare DENMARK (2020). New Hospital Construction-Future Hospitals in Denmark. Sustainable Hospitals, Odense.

[31] Ministry of Health (2018). A Coherent and Trustworthy Health Network for All: Digital Health Strategy 2018–2022. https://sum.dk/Aktuelt/Publikationer/A-Coherent-and-Trustworthy-Health-Network-for-All.aspx.

[32] Meister S., Burmann A., Deiters W., Urbach N., Röglinger M. (2019). Digital Health Innovation Engineering: Enabling Digital Transformation in Healthcare: Introduction of an Overall Tracking and Tracing at the Super Hospital Aarhus Denmark. Digitalization Cases.

[33] Alexander Geissler (2021). Instrument zur Evaluierung des Reifegrades der Krankenhäuser Hinsichtlich der Digitalisierung: DigitalRadar Krankenhaus Konsortium; Health Economics, Management and Policy No. 1. http://www.alexandria.unisg.ch/265296/.

[34] Hesser A. (2022). Erste Ergebnisse des Digitalradar: „Solide“ Digitalisierung in den Kliniken. Kma.

[35] Herr A. (2008). Cost and technical efficiency of German hospitals: Does ownership matter?. Health Econ..

[36] Mattei P., Mitra M., Vrangbæk K., Neby S., Byrkjeflot H. (2013). Reshaping public accountability: Hospital reforms in Germany, Norway and Denmark. Int. Rev. Adm. Sci..

[37] Thye J., Hübner U., Hüsers J., Babitsch B. (2017). IT Decision Making in German Hospitals-Do CEOs Open the Black Box?. Stud. Health Technol. Inform..

[38] Alten G., Nürnberg V., Blum K., Heber R., Lohse C. (2021). Schleudersitz Krenkenhausgeschäftsführer: BDO/DKI-Studie 2021, Köln. https://www.bdo.de/getattachment/Insights/Weitere-Veroffentlichungen/Studien/Schleudersitz-Krankenhausgeschaftsfuhrer/DKI-Studie-2021.pdf.aspx.

[39] Dillman D.A., Tortora R.D., Bowker D. (1998). Principles for Constructing Web Surveys.

[40] Schleyer T.K., Forrest J.L. (2000). Methods for the design and administration of web-based surveys. J. Am. Med. Inform. Assoc..

[41] Eysenbach G. (2004). Improving the quality of Web surveys: The Checklist for Reporting Results of Internet E-Surveys (CHERRIES). J. Med. Internet Res..

[42] Bethlehem J. (2010). Selection Bias in Web Surveys. Int. Stat. Rev..

[43] Sheskin D.J. (2011). Handbook of Parametric and Nonparametric Statistical Procedures.

[44] Benninghaus H. (2007). Die Beschreibung der Beziehung zwischen einer nominalen und einer metrischen Variablen. Deskriptive Statistik.

[45] Beaulieu M., Bentahar O. (2021). Digitalization of the healthcare supply chain: A roadmap to generate benefits and effectively support healthcare delivery. Technol. Forecast. Soc. Chang..

[46] Kohl S., Schoenfelder J., Fügener A., Brunner J.O. (2019). The use of Data Envelopment Analysis (DEA) in healthcare with a focus on hospitals. Health Care Manag. Sci..

[47] Sætra H.S., Fosch-Villaronga E. (2021). Healthcare Digitalisation and the Changing Nature of Work and Society. Healthcare.

[48] Laukka E., Pölkki T., Heponiemi T., Kaihlanen A.-M., Kanste O. (2021). Leadership in Digital Health Services: Protocol for a Concept Analysis. JMIR Res. Protoc..

[49] Kraus S., Schiavone F., Pluzhnikova A., Invernizzi A.C. (2021). Digital transformation in healthcare: Analyzing the current state-of-research. J. Bus. Res..

[50] Konttila J., Siira H., Kyngäs H., Lahtinen M., Elo S., Kääriäinen M., Kaakinen P., Oikarinen A., Yamakawa M., Fukui S. (2019). Healthcare professionals’ competence in digitalisation: A systematic review. J. Clin. Nurs..

[51] Nadri H., Rahimi B., Lotfnezhad Afshar H., Samadbeik M., Garavand A. (2018). Factors Affecting Acceptance of Hospital Information Systems Based on Extended Technology Acceptance Model: A Case Study in Three Paraclinical Departments. Appl. Clin. Inform..

[52] Gjellebæk C., Svensson A., Bjørkquist C., Fladeby N., Grundén K. (2020). Management challenges for future digitalization of healthcare services. Futures.

[53] Kokshagina O. (2021). Managing shifts to value-based healthcare and value digitalization as a multi-level dynamic capability development process. Technol. Forecast. Soc. Chang..

[54] Frick N.R.J., Möllmann H.L., Mirbabaie M., Stieglitz S. (2021). Driving Digital Transformation During a Pandemic: Case Study of Virtual Collaboration in a German Hospital. JMIR Med. Inform..

[55] Borsci S., Buckle P., Walne S., Salanitri D., Bagnara S., Tartaglia R., Albolino S., Alexander T., Fujita Y. (2019). Trust and Human Factors in the Design of Healthcare Technology. Proceedings of the 20th Congress of the International Ergonomics Association (IEA 2018).

[56] Burmann A., Tischler M., Faßbach M., Schneitler S., Meister S. (2021). The Role of Physicians in Digitalizing Health Care Provision: Web-Based Survey Study. JMIR Med. Inform..

[57] Laukka E., Huhtakangas M., Heponiemi T., Kanste O. (2020). Identifying the Roles of Healthcare Leaders in HIT Implementation: A Scoping Review of the Quantitative and Qualitative Evidence. Int. J. Environ. Res. Public Health.

[58] Baudier P., Kondrateva G., Ammi C., Chang V., Schiavone F. (2022). Digital transformation of healthcare during the COVID-19 pandemic: Patients’ teleconsultation acceptance and trusting beliefs. Technovation.

[59] Menon V., Muraleedharan A. (2020). Internet-based surveys: Relevance, methodological considerations and troubleshooting strategies. Gen. Psychiatr..

[60] Wu M.-J., Zhao K., Fils-Aime F. (2022). Response rates of online surveys in published research: A meta-analysis. Comput. Hum. Behav. Rep..

[61] Hoerger M. (2010). Participant dropout as a function of survey length in internet-mediated university studies: Implications for study design and voluntary participation in psychological research. Cyberpsychol. Behav. Soc. Netw..

